# Artificial Light at Night Promotes Activity Throughout the Night in Nesting Common Swifts (*Apus apus*)

**DOI:** 10.1038/s41598-019-47544-3

**Published:** 2019-07-30

**Authors:** Eran Amichai, Noga Kronfeld-Schor

**Affiliations:** School of Zoology, Faculty of Life Sciences, Tel Aviv University, Tel Aviv, Israel

**Keywords:** Zoology, Ecology

## Abstract

The use of artificial light at night (ALAN) is a rapidly expanding anthropogenic effect that transforms nightscapes throughout the world, causing light pollution that affects ecosystems in a myriad of ways. One of these is changing or shifting activity rhythms, largely synchronized by light cues. We used acoustic loggers to record and quantify activity patterns during the night of a diurnal bird – the common swift – in a nesting colony exposed to extremely intensive artificial illumination throughout the night at Jerusalem’s Western Wall. We compared that to activity patterns at three other colonies exposed to none, medium, or medium-high ALAN. We found that in the lower-intensity ALAN colonies swifts ceased activity around sunset, later the more intense the lighting. At the Western Wall, however, swifts remained active throughout the night. This may have important implications for the birds’ physiology, breeding cycle, and fitness, and may have cascading effects on their ecosystems.

## Introduction

Artificial light at night (ALAN) and its consequent light pollution is one of the most dramatic transformations caused by the industrial revolution^1,2^. Starting with gas lights in the early 1800s^3^, and more so with the adoption of electric bulbs in the late 1800s^4^, humans have increasingly introduced light into nighttime. Coupled with the global increase in human population and its expansion into more and more areas and environments, the last two centuries have seen an enormously significant increase in night light levels throughout the world and in almost all habitats^5,6^, with the rate of this light encroachment increasing annually^1,2^. Unlike other anthropogenic effects (e.g. CO_2_ emissions, temperature changes, etc.) ALAN has received less attention as an environmental issue and has remained relatively understudied until the beginning of the 21^st^ century^2^. It is now clear that ALAN affects numerous biological processes, both by providing illumination that hinders or enables physiological processes or behaviors^7–9^, and by interfering with the natural cues that synchronize lunar, circannual, and circadian rhythms^10–12^.

Birds are one of the groups most studied in the context of altered behavior patterns due to nighttime illumination. Several species of songbirds have been shown to display daytime behaviors at nighttime such as male singing, as well as more extra-pair copulation incidence in intensely illuminated areas^13^. While these effects may seem beneficial in part, their long term, direct and indirect consequences on both the individual and population level are unclear and potentially detrimental^14^.

The common swift (*Apus apus*) is a migratory bird that arrives in Israel early in the breeding season (mid-February) and establishes mostly urban breeding colonies. The urban environment provides a variety of suitable nesting sites: narrow, inaccessible hollows along building walls, that hold similar characteristics to the narrow crags and holes on cliffs and banks in which this species nests in natural habitats. Eggs are laid in March-April, incubated for 18–20 days, and the young fledge 34–43 days later (late May-June)^15^. Shortly after fledging the swifts leave their breeding colonies and migrate to currently unknown destinations. The species is assumed to sleep on the wing outside of the breeding season, with only occasional brief stops; some individuals have been recorded to fly non-stop for a period of ten months^16,17^. In contrast, breeding individuals roost nightly inside the nest after foraging during the day, occasionally returning to feed the nestlings^18^. Common swifts are a very vocal species, producing easily identifiable whistles while foraging and during social flights in the colony area at dawn and dusk^19^. While the function of these calls is unclear (may be purely social or may help coordinating movement within the group), they result in a clear acoustic pattern around the breeding colony of silence during the night and calls during the day, thus enabling the use of acoustic analysis to determine activity patterns in the colony. Two breeding colonies in Israel’s major cities were studied to some detail: one in Tel Aviv^20^, and the other at Jerusalem’s Western Wall^21^. While these studies agree with the general knowledge described above, an interesting observation was made in 2002 regarding the Western Wall colony. This site is an important religious site and is therefore illuminated by artificial lights throughout the night. The swifts in this colony were observed to leave their nests earlier in the morning and return later in the evening than their counterparts in Tel Aviv^21^.

In 2016 the lighting system in the Western Wall was replaced by a much more intense and widespread system. We hypothesized that the added illumination would result in a further extension of the swifts’ activity hours, and perhaps allow them to exploit a hitherto unavailable resource – moths and other nocturnal insects that can be attracted to the light. We used acoustic loggers to record *A. apus* activity in four breeding colonies differing in their nighttime light levels, and looked for moth-scales in fecal sacs obtained from the nests in order to check for dietary differences. We found that, surpassing our hypothesis, Western Wall swifts were active throughout the night while those of the other three colonies ceased their activity shortly after sunset. While we did not observe any attacks on moths by the night-active swifts, we found moth wing scales in fecal sacs of swifts from all three urban colonies.

## Results

We compared temporal activity patterns obtained from acoustic recordings between four breeding colonies differing in their level of ALAN exposure (see below and in methods): Jerusalem’s Western Wall (henceforth “Wall”), ALAN intensity 120 lx; Bar Ilan University (henceforth “BI”), ALAN intensity 3.85 lx; Tel Aviv (henceforth “TLV”), ALAN intensity 0.83 lx; and Hatira canyon (henceforth “Desert”), ALAN intensity 0.005 lx. We conducted visual observations and fecal sac microscopy to investigate moth consumption after sunset, in order to determine whether this prolonged activity enabled the swifts to exploit an obvious new resource. The entire study was conducted concurrently at all four sites during the pre-fledging period (see methods).

### Temporal activity patterns

**Figure 1:**
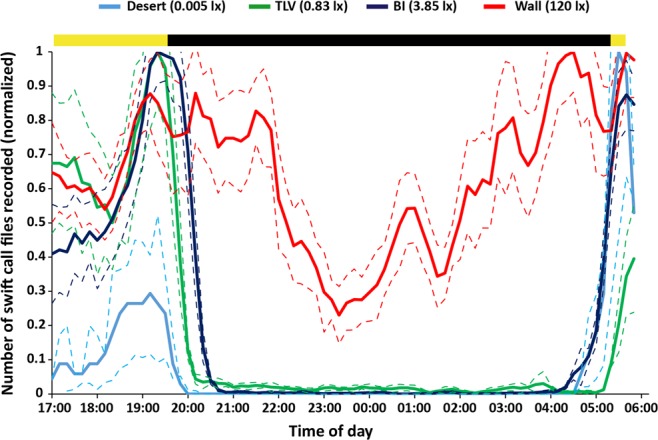
Temporal activity patterns. Swift activity as represented by acoustic recordings. Multi-night mean (3-period moving average ± se, represented by dashed lines of the same color) of the number of files containing swift calls per 10-minute bins. Data were normalized by dividing each time-bin value by the maximum time-bin value (of the same colony), to allow a comparative representation of the different sized colonies (e.g. “Desert” colony, which was much smaller and produced an order of magnitude fewer calls). The bar at the top depicts day/night (sunrise/sunset) cycle (yellow = day, black = night). At all three low-ALAN sites swift activity ended around sunset, while at the high-ALAN site (Wall) activity continued throughout the night. Note that the evening peak starts at the same time at all sites, but activity drops under lower-ALAN conditions first.

**Figure 2:**
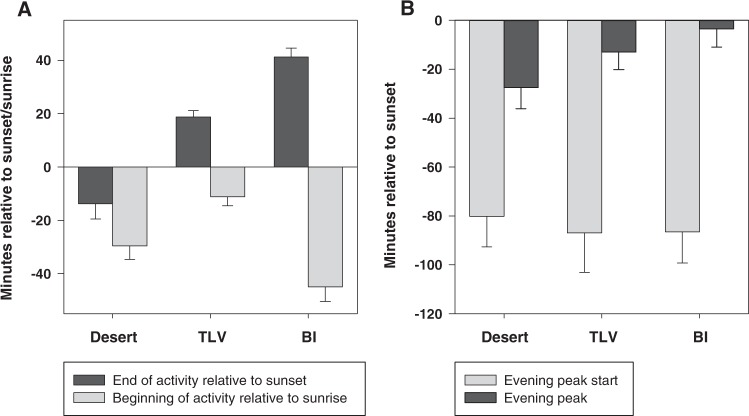
Timing of activity relative to sunset and sunrise. Average (mean ± se) time in minutes between sunset and the end of recorded activity (dark gray), and between sunrise and the first recorded activity (light gray) (**A**), at the three sites, ordered by ALAN level (Desert < TLV < BI). Zero represents sunset for end of activity and sunrise for beginning. The lower ALAN intensity, the earlier the swifts cease their daily activity. This is not the case in the beginning of activity: while the two artificial sites (TLV & BI) follow the pattern (less ALAN = later beginning), the natural site with the least ALAN shows an intermediate value and the swifts there began activity earlier than would be expected. (**B**) Average (mean ± se) time in minutes between sunset and the beginning (light gray) and peak (dark gray) of the evening activity peak. The beginning of the peak was defined as the time-bin with at least 50% more recordings than the previous time-bin. Peak was defined as the time-bin with the highest number of recordings before midnight. While the evening peak begins at about the same time at all three sites, its peak is reached later the more intense ALAN is.

We have recorded a total of 26,373 files containing swift calls (Wall: 16,670, 12 nights; BI: 5,801, 11 nights; TLV: 3,827, 10 nights; Desert: 75, 11 nights). To describe the temporal activity patterns we divided each night into ten-minute bins and counted the files containing calls in each bin. As swifts often fly in tight groups emitting overlapping calls it was impossible to count individual calls (see methods). Our results clearly show that while at the three lower-ALAN sites (BI, TLV, & Desert) activity followed a daily pattern – beginning around sunrise and ending around sunset, at the high-ALAN site (Wall) a high level of activity was maintained throughout the night (Fig. 1). At all sites there is a noticeable peak in activity around sunset. While this peak starts at the same time at all sites, its maximum and end differed among sites (Fig. 2), matching ALAN intensity: evening activity peak ended first in “Desert” colony (0.005 lx), then in “TLV” (0.83 lx), and later still in “BI” (3.85 lx); while in “Wall” (120 lx) activity never stopped and the peak stretched for four hours before activity dipped for a brief period before rising again around 02:00 (see also S1 for non-normalized, all nights’ data from all sites).

To ascertain the recorded patterns were not caused by anthropogenic noise, we checked for correlations between swift activity and background noise (see methods) during two full nights for each of the urban colonies (Fig. S2). The results of this analysis show that the correlation between the two is sometimes significant and sometimes non-significant (Table 1), but in all cases anthropogenic noise explains only a small portion of the variance in swift activity. In fact, some of this correlation can be explained by the recording settings of our recorders (see methods): a loud swift call that triggers the recorder can cause a quiet background noise to be recorded that would not have triggered the device by itself, and vice versa. No anthropogenic noise was present in the desert colony (see methods), and this colony was therefore excluded from this analysis.

**Table 1:** Correlations between anthropogenic noise and swift activity.

	Night 2 TLV	Night 7 TLV	Night 2 BI	Night 7 BI	Night 2 Wall	Night 7 Wall
R^2^	<0.001	0.06	0.21	0.047	0.056	0.19
t-statistic	−0.053	2.220	4.463	1.935	2.104	4.235
Lower 95% CI	−0.378	0.0354	0.559	−0.012	0.019	0.545
Upper 95% CI	0.358	0.652	1.459	0.830	0.688	1.514
P-value	0.957	0.029	<0.001	0.057	0.039	<0.001
Pearson coefficient	−0.006	0.247	0.456	0.217	0.235	0.437

Summarized results of two statistical tests: linear regression and Pearson correlation on two entire nights (night 2 and night 7) from each of the urban colonies. Plotted representation can be seen in Fig. S2).

Complete end of activity (i.e. time of last vocalization recorded each night) is also related to ALAN use level (Fig. 2A): in “desert”, activity ended 13.8 ± 5.3 minutes *before* sunset (mean ± se); in “TLV” 18.8 ± 2.2 minutes *after* sunset; and in “BI” 41.2 ± 3.3 minutes *after* sunset. Differences between all sites were significant – Kruskal-Wallis ANOVA on ranks followed by Dunn’s post-hoc test: N = 9/10/10, H = 24.12, df = 2, p < 0.001. At all three sites morning activity started before sunrise (Fig. 2A). While the two light-polluted sites followed the expected pattern – less ALAN equals later start, and the difference is statistically significant, at the natural site (“desert”) activity started unexpectedly early, with an intermediate value that is not statistically different to that of BI. One-way ANOVA followed by Tukey’s post-hoc test: N = 9/8/10, F = 11.95, df = 2, p < 0.001, TLV differs from Desert & BI, Desert & BI not statistically different.

Evening activity peak (Fig. 2B) started at the same time at all three sites (~85 minutes before sunset), with no statistical difference among the colonies – one-way ANOVA: N = 9/10/10, F = 0.07, df = 2, p = 0.93. It reached its peak following the same pattern as that of activity end (which was also the end of the evening peak): *i.e*. the higher the ALAN the later the end. however, though this trend is visible, it is not statistically significant – Kruskal-Wallis ANOVA on ranks: N = 7/10/10, H = 3.75, df = 2, p = 0.153.

### Foraging observations

Visual observations of foraging were conducted only in “wall” colony where nighttime activity took place. While large moths and swifts were often occupying the same space, the moths did not employ any escape maneuvers when swifts were flying towards them. For the most part the swifts ignored the moths and did not attack them (Table 2). The outcomes of observed attacks were not always clear – in one the moth was deflected back by the swift’s head, in two the swift missed the moth, and for the rest the outcome could not be determined. In no case was there a confirmed catch. In some cases, a swift collided with a moth, but for the most part swifts and moths seemed to ignore each other.

**Table 2:** Visual foraging observations.

	Observation 1	Observation 2	Observation 3
Number of swifts (average for 10 minute periods)	43	51	47
Number of moths (average for 10 minute periods)	4	7	10
Number of attacks (total for two hours)	2	2	3

Average numbers (per 10 minute intervals) of swifts and medium-large moths sharing the same space at the “Wall” colony, and total number of attacks throughout the entire two-hour observation period. All observations were carried out between 21:00 and 23:00 on non-consecutive nights.

### Fecal sacs analysis

We analyzed a total of 60 fecal sacs from the three urban colonies (20 from each) to check for moth scales (using a binocular microscope at 100x magnification, see methods). Lepidopteran scales (length range 150–300 µm) were present in fecal sacs from all three colonies (S3), with no apparent difference in their relative frequencies: TLV: 55% of fecal sacs contained moth scales; BI: 60%; Wall: 60%. The analyzed sacs were from several different nests within each colony. All the scales found lacked coloration and had various complex edge-shapes, suggesting that they belonged to moths rather than butterflies.

## Discussion

### ALAN and temporal activity patterns

Artificial lighting had a dramatic effect on the swifts’ daily activity patterns in “Wall” colony. This is a diurnal species, and while outside of the breeding season they may fly continuously and sleep on the wing^16,17^, during the breeding season the parents spend the night in the nest and activity in the colony ceases around sunset^18,20,21^. This was indeed the case in the other three colonies we studied that were exposed to low-to-medium ALAN intensity; however, in the fourth – “Wall” colony, which is exposed to extremely high-intensity ALAN, swifts’ activity at the colony continued throughout the night (Fig. 1). Exposure to ALAN is known to promote nighttime activity in diurnal species. In birds, nighttime activity increases mainly by shifting the beginning of activity earlier into the night (e.g.^13^). Examples of nocturnal species altering behavioral patterns in response to ALAN are numerous (e.g.^7,8,22^). Our results relate to a single population under each light condition, and thus represent a small sample size. In addition, since we studied the population level and have no knowledge of individual behavior we are cautious in assessing the generality of these results. However, to our knowledge this is the first report of a diurnal vertebrate species remaining active throughout the night in response to ALAN.

We cannot completely exclude the possibility that other anthropogenic factors affected activity patterns. The most obvious one would be noise. However, while human activity at “Wall” is non-stop, noise level at “TLV” is high as well as it is situated on a major road in a major city, and includes vehicles and loud music until well past midnight. Even in “BI”, inside a university campus, loud music and human speech were still present in the recordings more than three hours after the last swift had been recorded. Since the recorders were set to filter out the majority of anthropogenic noise, the real noise levels were actually much higher. Checking for correlations revealed that anthropogenic noise may have a small contribution to the emergence of the recorded activity patterns, though our results probably overestimate this effect (see methods and results).

In swifts, the length of the pre-fledging period is highly variable, and dependent on weather conditions: if the weather is bad and the parents cannot forage and feed the nestlings, fledging period lengthens^18^. If the swifts in our study use the night to forage and are thus able to shorten this period, we can speculate that two possible benefits may arise: 1. both fledglings and parents have more time available to exploit the seasonal bounty and prepare for migration; and 2. the parents may be able to migrate to different locations and exploit peaking resource availability for a second breeding cycle, a strategy known as ‘itinerant breeding’ that is employed by several migratory birds^23–25^. Our study did not reveal in what kind of behavior the “Wall” colony swifts engage at night: it may be purely social and not include foraging. However, we do not think this is the case – social flights (the so-called ‘screaming parties’) were performed next to the colonies, in three of the cases after sunset. In “BI” colony ALAN intensity is similar to that of a cloudy twilight (~3–5 lx)^26^, and yet activity ceases shortly after even though ALAN is stable. The continued activity at “Wall” colony suggests that higher light intensities are required, and since swifts rely on vision to detect their prey – small flying insects, only high intensity illumination will enable this. The fact that an earlier study of the same colony found that under the weaker lighting system the swifts did cease activity - albeit later than in Tel Aviv^20,21^ – strengthens the possibility that foraging was at least part of the nighttime activity.

Though it may seem that, at least in this case, light pollution has a bright side, this is not necessarily the case. Exposure to light at night, at much lower intensities than we record here, has been linked to long-term ill-effects such as disruption of reproductive cycles in birds^14^, and in humans many other health issues have been associated with chronic exposure to ALAN (e.g.^27^). It may be that though “Wall” swifts enjoy increased temporal niche for provisioning for their young, if measured over their lifetime their fitness is reduced. Further study is required to document and compare fledging period, migration dates and destinations, and possible itinerant breeding between the different colonies – an undertaking that is now becoming possible with the advent of lightweight tracking devices^16^.

Another interesting finding was that of the relative uniformity among sites in the timing of the beginning of the evening peak activity (see Figs 1 and 2B), and the differences between the sites in the time these peaks end (Fig. 2B). This peak represents a behavior called ‘screaming party’, the function of which is still unclear but seems to be purely social^18,28^. Both breeders and non-breeders perform fast flyovers around the colony, often crossing paths, all while emitting their characteristic screams (see S4). Presumably, at the end of this behavior the breeders enter the nests for the night while the non-breeders ascend to meet orientation demands prior to flight-sleep^29^. The onset of the ‘screaming parties’ seems to follow an internal, synchronized circadian rhythm anticipating the sunset, suggesting that the stretch of activity into the night where more light is available results from a masking effect, which does not influence the circadian clock (otherwise the clock controlled onset would change as well).

Light intensity is not the only factor that may influence activity patterns, and our results may be partially explained by other factors, either light-related (such as the direction the colony faces and its location in shaded vs. lighted spots^30^) and non-light-related, such as temperature and food availability^31^. Onset and ceasing of activity does not appear to be related to the light related factors: all nests are shaded; dusk activity is performed in the air and hence unrelated to light levels in the nest; activity onset is latest in the only eastwards-facing colony (“TLV”). The non-light-related factors, however, may have an effect on activity onset, especially in the “desert” colony: daily temperature range is wider in the desert with much higher maxima and lower minima compared to the urban colonies (max. 33.4 ± 1.3 °C, min. 17.8 ± 0.8 °C compared to max. 28.1 ± 1.1 °C, min. 21.4 ± 0.6 °C (mean ± se)). While nighttime minimum is only reached late and is anyway not sufficient to greatly reduce insect activity, the temperature in the nest may rise quickly and promote earlier onset. Perhaps more important is food availability: the desert holds a much lower food density and availability than the urban (Mediterranean) area. This likely means that longer commute and foraging bouts are necessary to feed the nestlings, driving the parents to start their activity as early as possible.

### Moth consumption

Previous studies did not include lepidopterans in swifts’ diet. As aerial insectivores, we expected that such an abundant food source as moths would offer a valuable new resource for the night-active swifts, just as they are for naturally nocturnal aerial insectivores such as bats. Our observations showed that the swifts do not try to hunt medium or large moths, and yet, moth remains have been found in equal frequency in all three urban colonies. These facts are not entirely surprising: the larger moths are well beyond the prey size swifts normally hunt^32,33^ and their small beaks may not be able to handle such prey; moth scales found in fecal sacs in our study were very small (150–300 µm) and were a challenge to spot at 100x magnification. Since there is a strong positive correlation between overall lepidopteran size and the size of their scales^34^, it appears the lepidopterans consumed were small as well and probably within the swifts’ normal prey size. Of the previous studies that studied swift diet only one reported the magnification used: 40x^33^ and it is possible that moth scales were present but overlooked. Since moth activity often begins at dusk and ends at dawn swifts have the opportunity to consume them in natural conditions, and it could be that our results do not represent a novel behavior, but simply reveal an undocumented one.

While we found no difference in moth consumption *frequency*, we have no knowledge of the *number* of consumed moths as our method was qualitative rather than quantitative (analysis was a presence/absence analysis of scales per fecal sac - see methods), and it may be that “Wall” colony swifts consume more moths than their conspecifics. Moreover, other insects are active at night – and drawn to the lights – so it cannot be ruled out that “Wall” swifts are indeed exploiting a resource unavailable to other swifts.

Interestingly, the medium-sized moths observed at this site (*Noctua pronuba*) are a tympanic species that perform evasive maneuvers in response to bat echolocation signals^35^. While the 2^nd^ and 3^rd^ harmonics of the swift’s calls are high enough in frequency to elicit such a response (see S4), most of the energy is concentrated in the fundamental and 1^st^ harmonic. At these frequencies the moths’ auditory nerves are least sensitive^35^, which could explain the lack of response. Should a larger aerial insectivore extend its activity into the night in response to ALAN, it will find an abundant prey that has not evolved the proper defense mechanisms.

### Summary

We found that while both medium and relatively high levels of ALAN (0.83 lx and 3.85 lx, respectively) caused common swifts to slightly extend their activity hours into the night in a breeding colony, swifts in a breeding colony exposed to very intense ALAN (120 lx) continue their activity throughout the night. This dramatic shift in activity patterns may have important and unknown implications for the swifts, as well as for their local ecosystem^11,12^.

## Methods

### Study sites

We chose four breeding colonies for the study, differing in the level of ALAN they are exposed to. Three of these are urban colonies while the fourth is a remote desert colony. The study was performed at a late stage of the breeding cycle – after hatching and before fledging, when chick food consumption is at its peak. This was done at all colonies concurrently between 07/05/2018 and 22/05/2018. While the three urban colonies are highly synchronized in their breeding onset, there is less information about the less accessible desert location, and breeding there may begin at a slightly different time. Following is a description of each colony. Wall, 31°46′N, 35°14′E: an urban colony in Jerusalem’s old city composed of about 90 pairs. This is a Jewish religious site and is the country’s most visited site. Human activity in the site is around the clock as is the artificial illumination. The birds nest in the holes between the stones comprising the wall, and perform social and foraging flights above the visitors’ open floor. The nests themselves are inaccessible, as only holes higher than human reach are utilized. ALAN intensity: 120 lx (see below for measurements details). BI, 32°04′N, 34°50′E: an urban colony inside Bar Ilan campus, in Ramat Gan – part of the Dan metropolitan area (Israel’s largest metropolitan complex). The colony is composed of about 200 pairs nesting in downwards-facing hollows that are part of some of the buildings’ architecture. Unlike in the Wall colony they are spread over several buildings covering a greater area and separated from each other, and facing northwards or eastwards. ALAN intensity: 3.85 lx. TLV, 32°04′N, 34°46′E: an urban colony in a high-rise building situated on a busy intersection next to Tel Aviv’s seaside promenade. The colony is composed of about 100 pairs nesting in downwards-facing hollows, primarily on the side of the building facing the quieter street (eastern face). ALAN intensity: 0.83 lx. Desert, 30°56′N, 35°03′E: a natural colony in a canyon cliff-face in a remote desert location. This is a mixed colony of *A. apus* and *A. pallidus*, comprising of about 10–15 pairs of each species nesting in holes in the cliff facing roughly southwards. ALAN intensity: 0 lx (environmental measurement: 0.005 lx, the lowest, natural background light value, with no added artificial light).

### Artificial light measurement

ALAN was measured once for each site, all measurements performed during the same night – a moonless, cloudless night (15/05/2018). The measurement was done at the location of the microphone (see below) after 21:05 (end of astronomical twilight). We used CEM DT-1308 light meter (ATP Instrumentation, UK) to measure visible light to an accuracy level of 0.001 lx. Sensor was held 100 cm above ground and pointing upwards. Performing the measurement in moonless, cloudless conditions provided a value consisting almost entirely of ALAN, including both direct and skyglow, as well as the low contribution of starlight. We did not try to achieve ALAN measurements across the entire area covered by the birds for several reasons: an averaged value would be just as informative as a single value, provided it was measured at a well-chosen point; a grid of values will be uninformative since our activity measurements were acoustic and therefore included no spatial information; the location of the microphone was chosen based on preliminary observation was (except for “desert”, see limitations below) the point below the most dense evening activity. We therefore measured ALAN at the location of the microphone to document its level at the location of peak activity.

### Acoustic activity recordings

*A. apus* approach their nests for feeding and sleeping while performing complex, often group circling flights, during which they produce intense scream-like calls that can be heard clearly and which are easily recognizable^19^ and measured (see S4). While during the day most of the foraging can be done far from the colony, towards evening the activity is concentrated around the colony, ending when the swifts enter their nests for the night and stop vocalizing. In the morning (typically before sunrise) activity starts and vocalizing resumes near the colony before the birds move away for the day. Thus recording acoustic activity in the colony provides a simple, quantifiable means to record the end and beginning of swift activity.

We used automatic acoustic loggers (SM4, wildlife acoustics, Inc., USA) positioned at the colony to record swifts’ calls. The loggers were programmed to record between 17:00 (circa 2.5 hours before sunset) and 06:00 (circa 20 minutes after sunrise) with a triggering threshold of 12 dB, no high-pass filter, sampling rate was set to 192 kHz. Recorded file length was limited to 8 seconds to facilitate analysis (see below). In the three urban sites (BI, TLV & Wall) the logger was positioned opposite the nests, 20–50 meters from the closest nest and facing the area in which the near-colony flights are performed. In the desert site the terrain did not allow such proximity as the nests are situated atop an inaccessible cliff. The recorder was thus set on the top of the opposite cliff, across the canyon (distance about 130 m). The combined effect of the much smaller colony size and the larger distance between recorder and nests resulted in fewer recordings by an order of magnitude. In the results we therefore plot normalized results of all sites (see Fig. 1), but though numerically they mismatch the rest of the sites, this does not affect the recorded activity temporal pattern. While it is possible that this also increases the rate of false negative detection, the acoustic conditions in “desert” were ideal, with very little wind and other background noise, and the combination of high intensity and low frequency of the calls means atmospheric attenuation should not be great enough to prevent calls from being recorded.

We have recorded a total of 26,373 files containing swift calls (Wall: 16,670, 12 nights; BI: 5,801, 11 nights; TLV: 3,827, 10 nights; Desert: 75, 11 nights).

### Acoustic analysis

We used SASLab Pro software (Avisoft Bioacoustics, Inc., Germany) to analyze the recordings. We used an automatic frequency-amplitude contours cross-correlation feature to detect and identify swift calls within the often background-noise-rich recordings. For this approach the first stage was to provide the software with over a hundred templates of manually identified swift calls (see S4) and a similar number of non-swift “trash” (anthropogenic noise or non-swift bird calls). The software then compares each detected call to all templates and classifies them to either “swift”, “trash”, or unknown class depending on the cross-correlation value. All analysis is performed on the calls spectrograms (FFT = 512, overlap = 75%, window: hamming). We manually validated the analysis around three potentially confusing or important times in each day: activity end, activity start, and activity peaks. We furthermore manually analyzed 900 files (300 files from each urban colony) and compared the results of this analysis to the automatic classification results. Based on this comparison we estimate the automatic classification error rate as ~2% for false-positive and ~5–10% for false-negative. We used corrected values for analysis.

Since often a large number of individuals are flying and calling at the same time and at close vicinity, it is many times difficult to separate individual calls when many are overlaid. We therefore used the number of files containing swift calls rather than the number of calls as an indicator to activity, resulting in a smoothing of activity in an eight-second window (each file length). We then divided the overall time into ten-minute bins, thus further smoothing the results.

To verify that activity patterns were not the result of anthropogenic noise, which is often correlated with ALAN, we manually analyzed two full nights from each of the urban colonies and counted the files containing anthropogenic noise. We then compared anthropogenic noise patterns to swift activity patterns (see results and Fig. S2). It is important to note that our recording settings and microphone specifications were optimized for swift calls detection. Therefore, not all anthropogenic noise was equally recorded, and human speech and music were more easily recorded than vehicle noise. While correlation between anthropogenic noise and swift activity was not high, it is likely lower than shown by our results, since the recording settings produce some correlation as an artifact: once the intensity threshold has been crossed the device records for eight seconds regardless of intensity. As a result, if a loud swift call triggers the device it will also record weak anthropogenic noises that would otherwise not be recorded, and vice versa. In the desert colony no anthropogenic noise was expected, and indeed none was recorded, resulting in this colony’s exclusion from the analysis. While natural background noise may have an effect on activity, its intensity in the desert colony was too low to trigger our recorder.

### Statistical analysis

We performed statistical analyses on four activity temporal measurements: 1. End of activity, defined as the time-bin with the last recording before midnight (excluding isolated nighttime recordings). 2. Start of activity, defined as the first time-bin with recordings that is followed by other time-bins with recordings (after midnight). 3. Beginning of evening peak, defined as the time-bin with a minimum of 50% increase in activity (50% more recordings than the previous time-bin), and 4. Evening peak, defined as the time-bin containing the highest number of recordings before midnight.

We used one-way ANOVA tests followed by Tukey post-hoc test on normally distributed data, and Kruskal-Wallis one-way ANOVA on ranks followed by Dunn’s method post-hoc test on non-normally distributed data. Statistical analyses were done using Sigmaplot 12 (Systat software, Inc., USA) software. Figures were made using Sigmaplot 12 and Excel 2016 (Microsoft, Inc., USA).

### Foraging observations and fecal sacs analysis

To investigate whether swifts have started to consume moths in the site where post-sunset activity exists we used two methods: I. on-site observations (only at the wall site): a total of six hours of visual observations were carried out at “wall” colony, three two-hours periods in non-consecutive nights between the hours of 21:00 and 23:00. Observations were from an elevated part of the open floor in the site, with unobstructed view of the entire site. For each ten-minute period the numbers of swifts flying, visible moths, and the number and outcome of attacks were counted. This method is of course limited to relatively large moths only, of which several were collected and identified as *Noctua pronuba* (family: Noctuidae). II. Fecal sacs analysis (at all sites excluding “desert”): This second method was qualitative rather than quantitative (the results comprising presence/absence data only with no information about the quantity of individual moths consumed), but did not exclude small moth species. Lepidopterans wings and bodies are covered with minuscule chitinous scales that are not digestible and are easy to find in feces (^36^ and see S3). Since lepidopterans are not normally part of *A. apus* diet^32,33^, it is safe to assume that scales found in feces of swifts that are active after sunset originate in moths. 20 fecal sacs were collected from within or under the 3–4 nests in each colony for analysis. We collected fecal sacs only after fledging because sampling was limited by “Wall” restrictions – due to the religious importance and political sensitivity of the site (Jerusalem’s Western Wall) we did not have free access to the nests but were limited to a single 15-minutes access in the year (6 weeks after fledging). From the “desert” colony no fecal sacs were collected due to the physical inaccessibility of the nests. Each sac was crushed manually an examined under a binocular microscope (100x magnification). We calculated the relative frequency of moth scales in fecal sacs and compared that between the colonies.

## Supplementary information

Supplementary Information
