# Supplementary material for: Artificial Light at Night Promotes Activity Throughout the Night in Nesting Common Swifts (Apus apus)
Source: Sci Rep. 2019 Jul 30;9:11052. doi: 10.1038/s41598-019-47544-3 (PMC6667432; doi:10.1038/s41598-019-47544-3)
Supplement: Supplementary file 1 — Supplementary Information [file 41598_2019_47544_MOESM1_ESM.pdf]

Artificial Light at Night Promotes Activity Throughout the Night in Nesting Common Swifts (*Apus apus*)

Eran Amichai[1] & Noga Kronfeld-Schor[1]

[1] School of Zoology, Faculty of Life Sciences, Tel Aviv University

Supplementary materials

Supplementary figures S1, S2, S3, S4

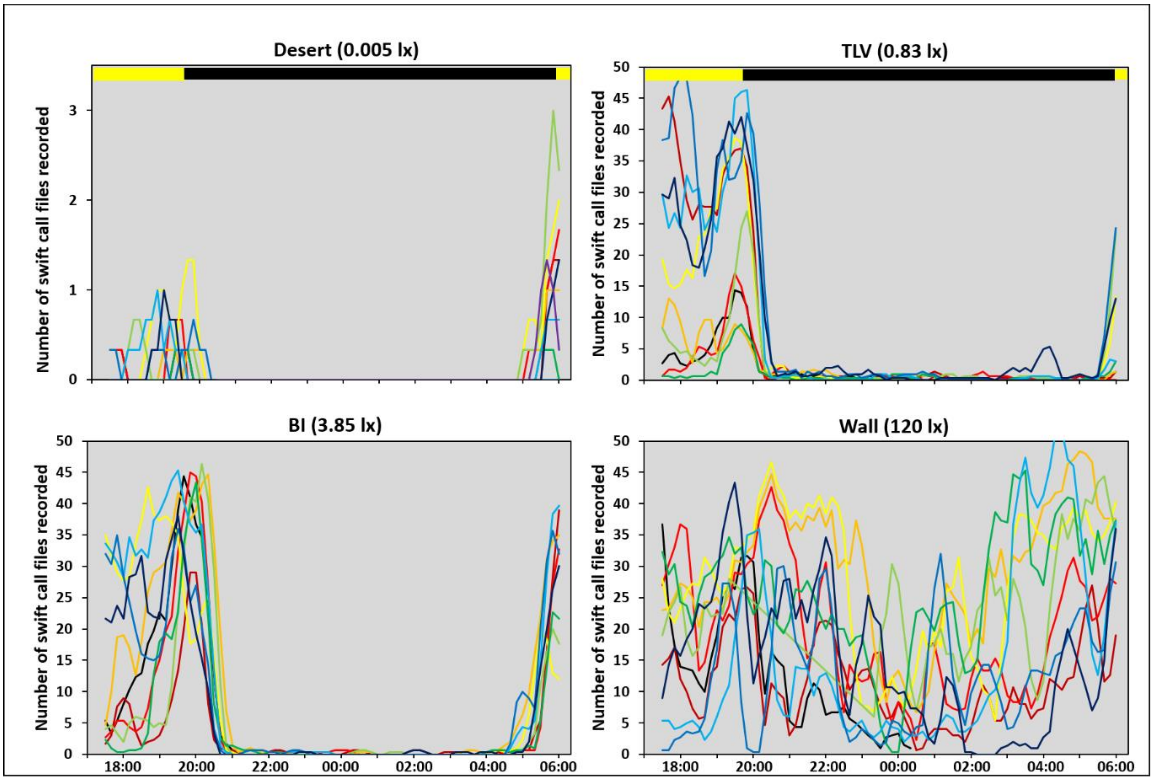

Figure S1: Nightly temporal activity patterns. Swift activity as represented by acoustic recordings. Each site is depicted in a different panel, each night in a different color. Data presented are 3-period moving average of the number of files containing swift calls per 10 minute bins. The bar at the top depicts day/night cycle (yellow=day, black=night). During all nights, in all three low-ALAN sites swift activity ended around sunset, while in the high-ALAN site (Wall) activity continued throughout the night.

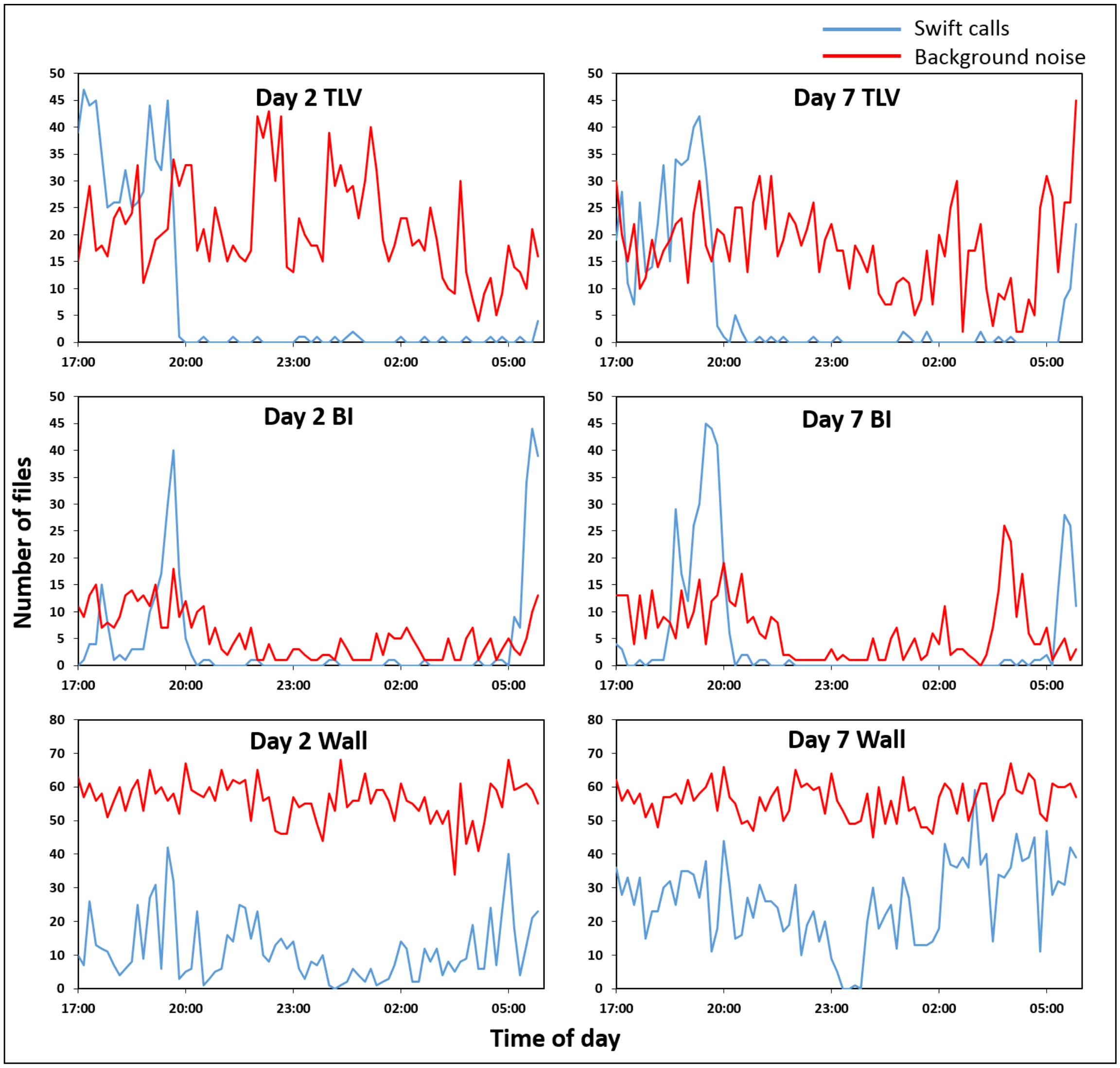

Figure S2: Anthropogenic noise and swift activity. Number of files containing anthropogenic noise (red line) overlaid upon the same night's swift activity (blue line - number of files containing swift calls). Analysis of two entire nights from each urban colony. In "TLV" anthropogenic noise consisted at roughly the same level throughout most of the night (with a decline starting after midnight), while swift activity ended shortly after sunset. In "BI" noise level was lower and the night was mostly quiet, however swift activity ended while noise level was still high. In "Wall" noise level was consistently high throughout the night, while nightly swift activity fluctuated. Note that "noise level" only depicts number of files and NOT noise intensity. The high noise level (large number of files containing noise) does not imply necessarily the environment is noisier: the recording filter optimal for swift call triggering also favors human speech, which constitutes much of the noise in that colony, over vehicle noises that contribute much of the noise in "TLV". In all cases noise intensity was either similar or weaker than swift calls.

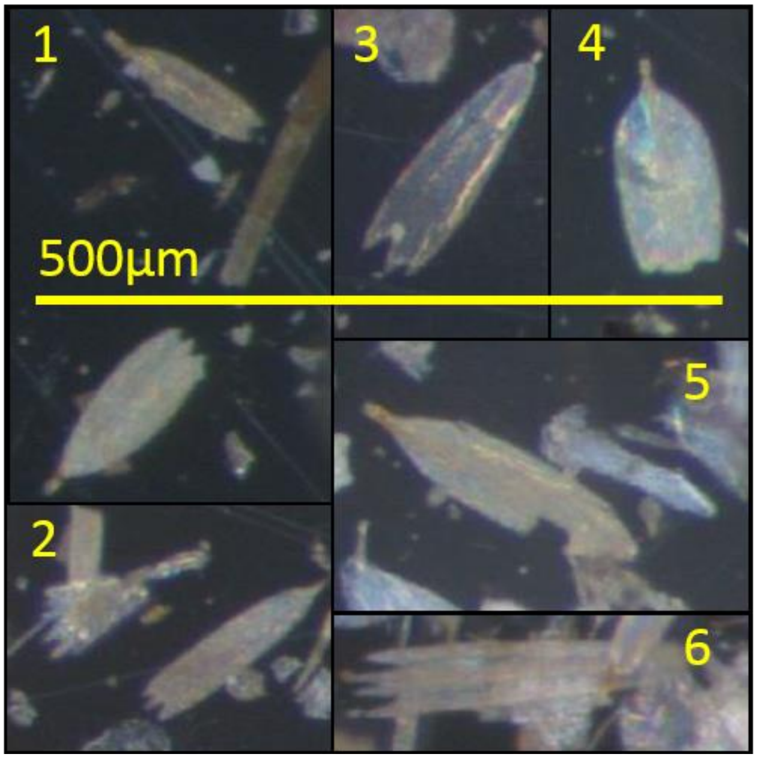

Figure S3: Moth scales in fecal sacs. An assortment of moth scales found in fecal sacs from all colonies: 1-2: Wall, 3-5: BI, 6: TLV.

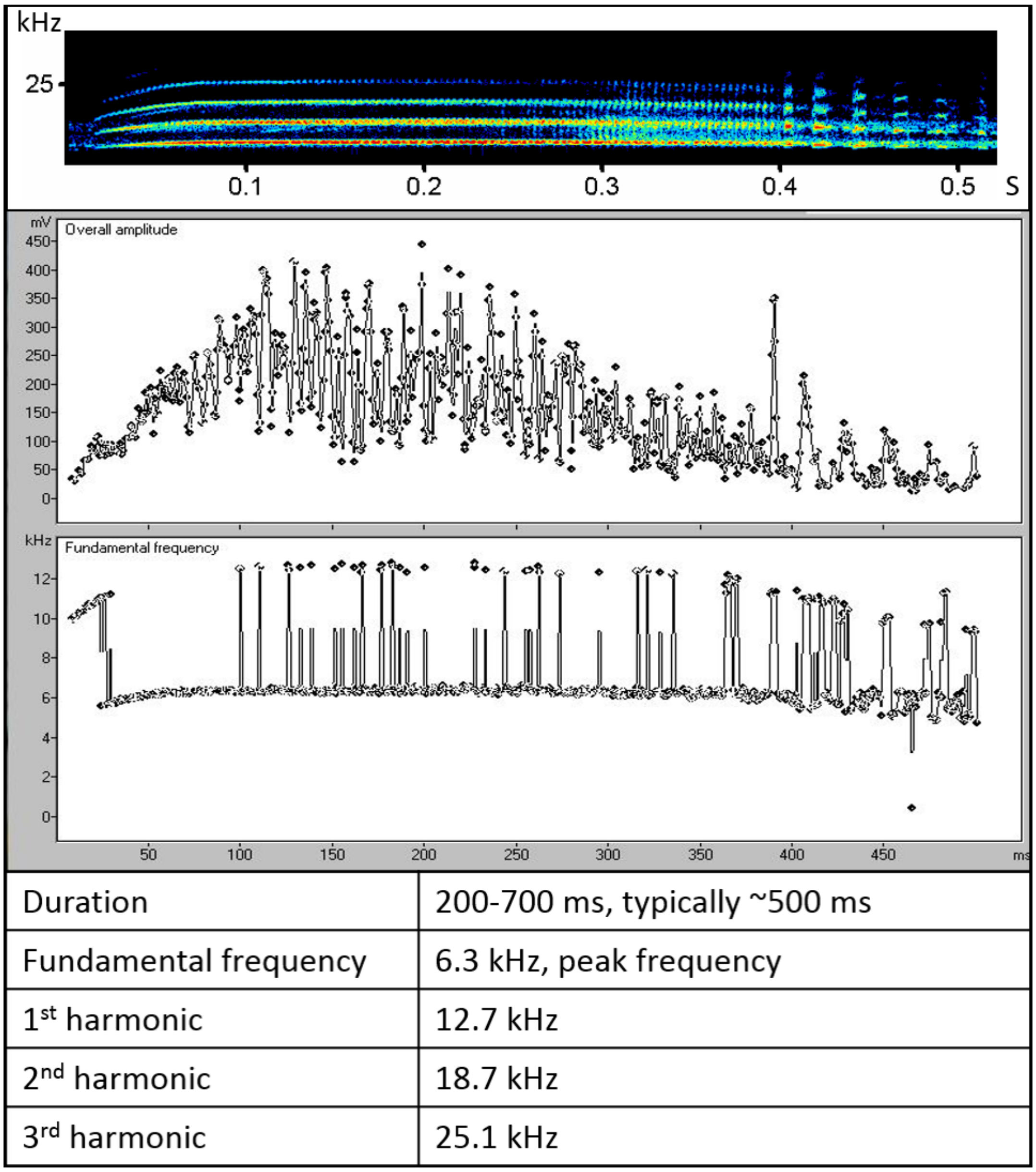

Duration	200-700 ms, typically ~500 ms
Fundamental frequency	6.3 kHz, peak frequency
1st harmonic	12.7 kHz
2nd harmonic	18.7 kHz
3rd harmonic	25.1 kHz

Figure S4: Acoustic characteristics of common swift scream call. *Top*: a spectrogram of a typical call. Notice the multi-harmonic structure of the call and the trill at its end. Though there is considerable variability in the calls' duration, they are usually around 500 ms in length. The harmonic structure is quite stable, with the fundamental frequency and 1st harmonic containing the most energy. *Middle*: the same call's frequency-amplitude contours template (amplitude above and frequency below) – one of ~100 used for automatic classification. *Bottom*: major acoustic parameters of the calls.
